# Granisetron versus Ondansetron: Comparison of 5HT_3_ antagonists in preventing spinal anaesthesia induced hemodynamic instability in obstetric patients

**DOI:** 10.12669/pjms.38.7.5585

**Published:** 2022

**Authors:** Maheen Wahid, Shabana Ali, Bilal Yasin, Kulsoom Farhat, Mudassar Noor, Fatima Tassadaq Syed

**Affiliations:** 1 Dr. Maheen Wahid, MBBS, MPhil (Pharmacology), Department of Pharmacology, Army Medical College, National University of Medical Sciences, Rawalpindi, Pakistan; 2 Dr. Shabana Ali, MBBS, MPhil, FCPS. Department of Pharmacology, Army Medical College, National University of Medical Sciences, Rawalpindi, Pakistan; 3 Dr. Bilal Yasin, Department of Anaesthesia, Combined Military Hospital, Rawalpindi, Pakistan; 4 Dr. Kulsoom Farhat, Department of Pharmacology, Army Medical College, National University of Medical Sciences, Rawalpindi, Pakistan; 5 Dr. Mudassar Noor, Department of Pharmacology, Army Medical College, National University of Medical Sciences, Rawalpindi, Pakistan; 6 Fatima Tassadaq Syed, Department of Anaesthesia, Combined Military Hospital, Rawalpindi, Pakistan

**Keywords:** Bradycardia, Granisetron, Hypotension, Ondansetron, Spinal Anaesthesia

## Abstract

**Objectives:**

To evaluate and compare the Ondansetron and Granisetron in preventing spinal anaesthesia induced hemodynamic instability in obstetric patients

**Methods:**

The comparative analytical study was conducted at Combined Military Hospital, Rawalpindi, from September to October, 2021. One hundred and twenty pregnant women undergoing cesarean section, were enrolled in the study via non probability convenience sampling, and divided into three groups containing 40 participants each based on the type of antiemetic premedication they received, if any: Group N were those not requiring antiemetic premedication, Group O consisted of those given ondansetron 4mg, and Group G had those receiving 3mg granisetron, 15 minutes prior to administration of spinal anaesthesia. Systolic blood pressures and heart rates were recorded before and at multiple intervals after spinal anaesthesia was administered. Episodes of hypotension and bradycardia were recorded. Requirement of phenylephrine and atropine as rescue drugs was recorded for each participant.

**Results:**

There was a statistically significant difference in incidence of hypotension among the three groups (*p* value <0.001), with both drugs being superior to the control group (*p* value <0.001 for both), and 3mg granisetron being superior to 4mg ondansetron (*p* value <0.001). As for incidence of bradycardia, ondansetron and granisetron were superior to control group (*p* value 0.03 and <0.001 respectively), but there was no significant difference between the two drug groups (*p* value 0.094).

**Conclusion:**

High dose granisetron (3mg) is superior to low dose ondansetron (4mg) in preventing hemodynamic fluctuations induced by spinal anaesthesia.

## INTRODUCTION

Use of 5HT_3_ receptor antagonists is among the novel therapeutic approaches being employed to battle with the notorious hemodynamic complications associated with spinal anaesthesia: hypotension and bradycardia.^1^ The antiemetic drugs, ondansetron and granisetron, are two such commonly used 5HT_3_ receptor antagonists.

The deteriorating hemodynamic aftermath of spinal anaesthesia is experienced by 33% patients for hypotension, and 13% for bradycardia.^2^ However, these figures skyrocket to 75% in patients undergoing cesarean section, owing to the added compression of the vasculature by the gravid uterus^3^. The hemodynamic instability carries massive morbidity as it can present with headache, nausea and vomiting when mild, but can lead to altered sensorium and even cardiac arrest and death if severe and left untreated. Similarly, fetal distress is precipitated as hemodynamic status worsens.^3^

Current therapeutic strategies adopted to combat this complication are use of rescue medications, which include vasopressors (phenylephrine and ephedrine) and atropine, all of which carry maternal and/or neonatal adverse effects of their own.^3^

Whereas intrathecal injection of the local anaesthetic agent itself causes cessation of the sympathetic outflow in the regions involved, leading to parasympathetic overdrive and hence fall in blood pressure and heart rate, this drop in the hemodynamic parameters causes activation of 5HT_3_ receptors present in the inferoposterior wall of the left ventricle. This leads to inhibition of the vasomotor center, further decreasing blood pressure and heart rate (The Bezold Jarish reflex).^4^ Hence, prophylactic blockade of these receptors will lower the incidence of hypotension and bradycardia, and ultimately lead to a decline in the requirement of the aforementioned rescue medications.^5^

Ondansetron is frequently utilized in anaesthetic and surgical practice for the prevention of post-operative nausea and vomiting, as it provides antiemetic effect around 3 hours^6^, with multiple dosing often required. Its role as pre anaesthetic drug for maintenance of hemodynamic status is recently being evaluated with promising results. It may be used at a dose of 4mg to 8mg^7^. However, lower doses of ondansetron are preferred as the risk of QT prolongation, although rare, escalates with higher doses, while the efficacy remains constant.^8^

Granisetron is another 5HT_3_ receptor antagonist that, in comparison to ondansetron, provides a stronger and longer antiemetic effect up to nine hours.^6^,^9^ It is commonly used in the prevention of chemotherapy induced nausea and vomiting. Its efficacy as premedication for preventing hemodynamic instability during anaesthesia has recently been elucidated. This potent 5HT_3_ receptor antagonist can be safely administered at a dosage of 1mg to 3mg, with dose dependent increase in efficacy, with minimal to no risk of cardiac adverse effects.^7^,^10^

This study aimed to evaluate the comparative effectiveness of granisetron and ondansetron in preventing the hemodynamic response associated with spinal anaesthesia in patients undergoing cesarean section.

## METHODS

The comparative analytical study was conducted from September to October 2021, in Combined Military Hospital (CMH), Rawalpindi, Pakistan. Approval was taken from the Ethics Review Committee at Army Medical College, National University of Medical Sciences (ERC/ID/123, on 7^th^ September, 2021), and at CMH (Serial Number: 175/6/21, on 3^rd^ September 2021). A sample size of 41 was calculated using reported incidence of hypotension^11^, via the World Health Organization (WHO) sample size calculator. However, a sample size of 120 was kept to improve the power of the study, with 40 participants in each group.

Eligible participants were enrolled via non probability convenience sampling, and were then further divided into three groups according to the premedication they received: Group N (n=40) received premedication with no antiemetic drug, Group O received 4mg ondansetron (IV), and Group G received 3mg granisetron (IV) as part of preanaesthetic medication, 15 minutes prior to administration of spinal anaesthesia. Pregnant females between the ages 18 to 50, with ASA Grade II or below who gave written informed consent, undergoing elective cesarean section. Patients having any contraindication to spinal anaesthesia, taking triptans, MAO inhibitors or SSRIs, having history of PIH, cardiovascular, renal, hepatic, neuropsychiatric diseases, alcohol or drug addiction, reaching anaesthetic level rise beyond dermatome T4 (based on skin test), or having intraoperative bleeding exceeding 1 liter were excluded from the study.

All participants were counselled preoperatively. A fasting state of eight hours was ensured. Demographic details, age, weight and gestational age were recorded. All patients were preloaded with 1000ml Ringer’s Lactate prior to the procedure. Noninvasive blood pressure monitoring, electrocardiogram and pulse oximetry, were used as monitoring techniques. Preanaesthetic medications were administered as per protocol. Baseline systolic blood pressure (SBP) and heart rate were recorded.

The anaesthetic technique was uniform for all participants. After being placed in sitting position, lumbar puncture was performed, and 10 to 15 mg of hyperbaric 0.5% bupivacaine was administered in the midline in intervertebral space between L3 and L4 using 25 gauge spinal needle. Patient was then placed in supine position.

15ml/kg/hour infusion of Ringer Lactate was administered throughout the procedure. Hemodynamic variables were recorded right after institution of spinal anaesthesia, then at regular intervals of two minutes for the first 10 minutes, and then of five minutes for the next 20 minutes.

Incidences of hypotension, defined as SBP more than 20% below the baseline, and incidence of bradycardia, defined as heart rate more than 20% below the baseline, were recorded for each patient.^7^ Five hundred micrograms of phenylephrine, and 0.5mg of atropine were administered as IV boluses to treat each episode of hypotension and bradycardia respectively. Total number of boluses used were recorded, and total dose administered was calculated for each patient.

Statistical analysis was done using SPSS 25. Normality of data was checked using the Shapiro Wilks test. Quantitative data was presented as mean with standard deviation, and median with interquartile range as appropriate. Kruskal Walis test, Mann Whitney U test, ANOVA followed by Post Hoc Tukey’s tests were employed as applicable. Qualitative variables were presented as frequencies and percentages, and analyzed using Pearson’s Chi Square test. Confidence Interval of 95% was used, and level of significance was set at *p* ≤ 0.05.

## RESULTS

Demographic details of participants in all three groups is presented in Table-I. Distribution of cases according to age, weight, duration of surgery, anaesthesia-to-incision time, blood loss and gestational age was statistically comparable.

**Table-I:** Clinical characteristics of participants in the three study groups.

Parameters	Group-N	Group-O	Group-G	p value
Age (years) (Mean±SD)	29.48±3.89	29.20±4.51	29.20±4.55	0.948
Weight (kg) (Mean±SD)	73.13±13.24	70.18±13.10	72.33±13.36	0.589
Duration of Surgery (minutes) (Mean±SD)	36.68±6.40	36.90±6.99	34.93±6.56	0.341
Anaesthesia-To-Incision Time (minutes) (Median (IQR))	7 (6-9)	7 (5-8)	7.5 (5-9)	0.112
Blood Loss (Ml) (Median (IQR))	225 (150-300)	200 (150 – 250)	200 (150-300)	0.234
Gestational Age (weeks) (Mean±SD)	338.10±1.22	37.73±1.18	38.35±1.12	0.056

**Fig.1:**
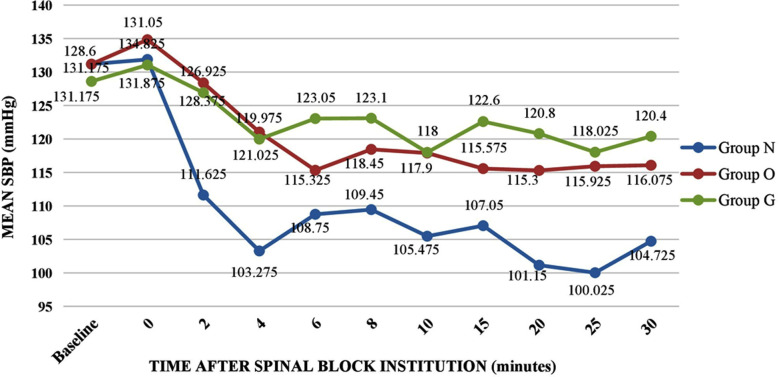
Variation of SBP among the three study groups.

**Fig.2:**
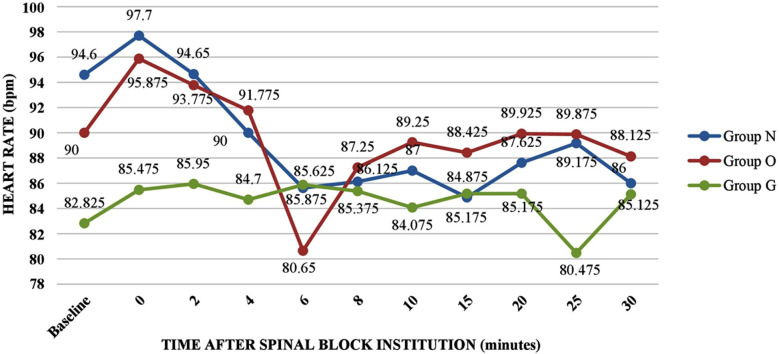
Variation of heart rate among the three study groups.

All three groups were statistically comparable in baseline SBP and heart rate. All groups showed a decrease in the hemodynamic parameters after institution of spinal anaesthesia. SBP remained higher for Group O and Group G as compared to Group N (Fig.1). However, there was no significant difference in mean heart rates (Fig.2) among the three groups.

There was statistically significant difference among incidence of hypotension among the three groups, with Group G showing the least hypotensive episodes. Table-II. There was significant difference among incidence of bradycardia between the control group and the study groups. However, there was no significant difference in bradycardiac episodes between Group O and Group G.

**Table-II:** Incidence of hemodynamic fluctuations (frequency & percentage) and median dose of rescue drugs (median with IQR) among the three study groups.

	Group			p value			

	Group N	Group O	Group G	Group N vs Group O	Group N vs Group G	Group O vs Group G	Group N vs Group O vs Group G
Incidence of Hypotension	36 (90%)	24 (60%)	7 (17.5%)	0.020	<0.001	<0.001	<0.001
Incidence of Bradycardia	24 (60%)	11 (27.5%)	5 (12.5%)	0.030	<0.001	0.094	<0.001
Dose of Phenylephrine (Median (IQR))	500 (300-600)	100 (0-300)	0 (0-0)	<0.001	<0.001	<0.001	<0.001
Dose of Atropine (Median (IQR))	0.5 (0-1.0)	0 (0-0.5)	0 (0-0)	0.008	<0.001	0.085	<0.001

The overall dose of phenylephrine and atropine required in each group is also displayed in Table-II, with statistically significant difference among the three groups, and the least dose of phenylephrine required in Group G. However, there was no significant difference in atropine consumption in between Group O and Group G, with both groups requiring lesser atropine than the control.

## DISCUSSION

Hemodynamic instability is a well-known and notorious complication associated with spinal anesthesia, especially in cesarean section. It presents as a major clinical challenge as the deteriorating hemodynamic status is troubling not only for the patient, but also for the anaesthetists and surgeons, with life threatening outcomes in the mother and child. Episodes of hypotension and bradycardia are countered with multiple pharmacological techniques, which include the use of phenylephrine and atropine. Unfortunately, such therapeutic approaches bring more harm than good, and bear numerous adverse effects of their own, while augmenting the financial burden on the patient. Hence, this hemodynamic disaster presents as a grave dilemma in the clinical setup.

The degenerating hemodynamic integrity is partly attributed to the 5HT_3_ receptors in the heart. Blockade of these receptors is among the novel therapeutic approaches being developed to battle with this problem. Two 5HT_3_ antagonists - ondansetron and granisetron – are currently under investigation in this regard. This study is the first to compare low dose ondansetron (4mg) with high dose granisetron (3mg) in preventing hemodynamic fluctuations, as well as minimizing requirement of rescue medications.

In the present study, pretreatment with 4mg ondansetron restored blood pressures to normal values as opposed to the control group. Incidence of hypotension and bradycardia was lower in the ondansetron group, with decreased consumption of phenylephrine and atropine when compared to the control group. Similar findings were demonstrated in a study conducted by Shabana and her colleagues in 2018^12^ where 100 parturients were divided into two groups: one receiving 4mg ondansetron while the other received 10ml of normal saline, five minutes prior to spinal block. Also, Xiao with his friends in 2017 showed that 4mg ondansetron reduces the need of phenylephrine to manage hypotensive episodes^13^. Findings of our study are in agreement with this. Our results also concur with a study conducted by Wang et al. in 2014^14^, where 150 parturient women were divided into five groups: one receiving 5ml normal saline, and the remaining groups receiving 2mg, 4mg, 6mg and 8mg ondansetron each. This study concluded that 4mg ondansetron is the most optimal dose for prevention of spinal anaesthesia induced hemodynamic derangement, which is comparable with our findings.

In the present study, 3mg granisetron, when compared to the control group, successfully reinstated the blood pressures and heart rates to preanaesthetic values. Hypotensive and bradycardiac episodes, along with rescue drug requirement was significantly reduced as opposed to the control group. Abdalla and Ammar conducted a study in 2017 which produced similar findings, with 54 patients undergoing various infraumblical surgeries being divided into two groups: one receiving 1mg granisetron and the other receiving 5ml normal saline, 5 minutes preanaesthesia.^15^ Another study in 2015 by Eldaba and Amr enrolled 200 obstetric patients and concluded that premedication with 1mg granisetron effectively prevents spinal anaesthesia induced hemodynamic derangement and vasopressor usage.^16^ Similar results were also exhibited in a study by Chatterjee and colleagues in 2020, where 200 obstetric patients were enrolled and divided into one group receiving 1mg granisetron 10 minutes preanaesthesia, and other group kept as control.^17^ Granisetron successfully lowered the incidence and extent of hypotension in this study, along with vasopressor utilization.

Our study concluded that granisetron is significantly superior in preventing spinal anaesthesia induced hypotension, along with decreasing phenylephrine consumption. However, both drugs comparably prevent bradycardia and lower atropine requirement. Hence, the important finding of the present study is the increased efficacy of granisetron in prevention of hemodynamic responses associated with spinal block. Aksoy and his colleagues in 2021 also compared both drugs in 80 obstetric patients and deduced that ondansetron and granisetron are equally effective^1^. A similar study by Khalifa in 2015 with 80 parturients comparing 1 mg granisetron with 4 mg ondansetron concluded that while both drugs are effective in prevention of hemodynamic fluctuations, ondansetron is superior.^18^ These findings partially contradict results of the present study, which could be due to different doses of drugs used in our investigation.

Granisetron shows dose dependent action. Increasing the dose enhances efficacy of the drug. This was demonstrated in a study using prophylactic granisetron in two different doses to prevent post spinal shivering.^10^ A total of 90 patients undergoing septorhinoplasty under general anaesthesia were divided into two groups: one receiving 1mg granistertron and the other receiving 3mg. This study showed that a higher dose of granisetron (3mg as compared to 1mg) more effectively reduces post spinal shivering. A similar study in 2021 also compared 3mg with 1mg granisetron in 244 patients undergoing cesarean section, and inferred that post spinal shivering, nausea and vomiting were significantly lowered with high dose granisetron.^19^ Thus action of granisetron is dose dependent. Our study uses a higher dose of the drug i.e. 3mg. This increased dose could be the possible reason for improved efficacy of granisetron in the present study, when compared to 4mg ondansetron.

Granisetron can safely be used in higher therapeutic doses, as it carries minimal to no risk of cardiac adverse effects. Ondansetron, however, carries the risk of QT prolongation with higher therapeutic doses, and is recommended to be used at a safer dose of 4mg, as efficacy is not dose dependent.^8^

Hence, the present study is the first to establish superiority of high dose granisetron (3mg) over low dose ondansetron (4mg) when used as premedication to prevent hemodynamic instability. Granisetron is more expensive than ondansetron, but in high dose it provides better and longer lasting antiemetic effect, ameliorates spinal anaesthesia induced hemodynamic responses and lessens vasopressor consumption. This justifies the higher cost of the drug, as the monetary load per patient will then be reduced.^20^

### Limitation of the study

This study enrolled 120 patients. A larger sample size may be used for more precise results.

## CONCLUSION

High dose granisetron (3mg) is superior to low dose ondansetron (4mg) in preventing hypotensive episodes induced by spinal anaesthesia. However, both drugs do not differ in their action in preventing bradycardia.

### Authors’ Contribution:

**MW:** Conceived, designed, collected data and did statistical analysis with interpretation & manuscript writing

**SA:** Project supervision right from its conception, and evaluated manuscript for important intellectual content

**BY & FTS:** Supervised the entire project in the hospital, revised the manuscript critically for final publication

**SA, MN & KF:** Did review and final approval of manuscript; , responsible for accuracy and integrity of data

## References

[1] Aksoy M, Dostbil A, Aksoy AN, Ince I, Bedir Z, Ozmen O (2021). Granisetron or ondansentron to prevent hypotension after spinal anesthesia for elective cesarean delivery:A randomized placebo-controlled trial. J Clin Anesth.

[2] Tatikonda CM, Rajappa GC, Rath P, Abbas M, Madhapura VS, Gopal NV (2019). Effect of intravenous ondansetron on spinal anesthesia-induced hypotension and bradycardia:A randomized controlled double-blinded study. Anesthesia, essays and researches.

[3] Lee JE, George RB, Habib AS (2017). Spinal-induced hypotension:Incidence, mechanisms, prophylaxis, and management:Summarizing 20 years of research. Best Pract Res Clin Anaesthesiol.

[4] Biricik E, Unlugenc H (2021). Vasopressors for the Treatment and Prophylaxis of Spinal Induced Hypotension during Caesarean Section. Turk J Anaesthesiol Reanimat.

[5] Kim HB, Lee JM, Choi ES, Lee SY, Ahn WS (2013). Effects of different kinds and different doses of 5-HT3 receptor antagonists on prevention of hypotension after spinal anesthesia:8AP6-2. Eur J Anaesthesiol.

[6] Goodman LS, Brunton LL, Hilal-Dandan R, Knollmann BC (2017). Goodman &Gilman's:The pharmacological basis of therapeutics.

[7] Aitkenhead AR, Moppett IK, &Thompson JP (2019). Smith and Aitkenhead's Textbook of Anaesthesia, Thompson JP, Moppett IK &Wiles MJ (Eds), Edinburgh. Churchill Livingstone/Elsevier.

[8] Attia ZM, AbdAllah MM (2020). A Comparative Study of Two Different Doses of Intravenous Ondansetron for Prevention of Post-spinal Anesthesia Shivering in Inguinal Hernia Repair Surgery. Zagazig Uni Med.

[9] Salajegheh S, Kuhestani S, Kermani MS, Taheri O, Bafghi NN (2019). Comparison of Ondansetron and Granisetron Effects for Prevention of Nausea and Vomiting Following Strabismus Surgery. Open Access Macedonian J Med Sci.

[10] Gargari RM, Anvari HM (2017). Effect of Different Doses of Granisetron on Preventing Postoperative Shivering in Patients undergoing Septorhinoplasty under General Anesthesia. Adv Biosci Clin Med.

[11] Anisha,Narayan S (2020). A comparative study between intravenous Ondansetron and Granisetron in attenuation of hypotension during spinal anaesthesia in patients undergoing caesarean section. Indian J Clin Anaesth.

[12] Shabana AA, Elkholy NI, Mohamed AM, Hamid MI (2018). Effect of ondansetron on hypotension and bradycardia associated with spinal anesthesia during cesarean section. Menoufia Med J.

[13] Xiao F, Wei C, Chang X, Zhang Y, Xue L, Shen H (2019). A Prospective, Randomized, Double-Blinded Study of the Effect of Intravenous Ondansetron on the Effective Dose in 50% of Subjects of Prophylactic Phenylephrine Infusions for Preventing Spinal Anesthesia–Induced Hypotension During Cesarean Delivery. Anesth Analg.

[14] Wang M, Zhuo L, Wang Q, Shen MK, Yu YY, Yu JJ (2014). Efficacy of prophylactic intravenous ondansetron on the prevention of hypotension during cesarean delivery:a dose-dependent study. Int J Clin Exper Med.

[15] Abdalla W, Ammar MA (2017). Systemic granisetron can minimize hypotension and bradycardia during spinal anesthesia in patients undergoing elective lower-abdominal surgeries:A prospective, double-blind randomized controlled study. Ain-Shams J Anaesthesiol.

[16] Eldaba AA, Amr YM (2015). Intravenous granisetron attenuates hypotension during spinal anesthesia in cesarean delivery:A double-blind, prospective randomized controlled study. J Anaesthesiol, Clin Pharmacol.

[17] Chatterjee A, Gudiwada B, Mahanty PR, Kumar H, Nag DS, Ganguly PK (2020). Effectiveness of Granisetron in Prevention of Hypotension Following Spinal Anaesthesia in Patients Undergoing Elective Caesarean Section. Cureus.

[18] Khalifa OS (2015). A comparative study of prophylactic intravenous granisetron, ondansetron, and ephedrine in attenuating hypotension and its effect on motor and sensory block in elective cesarean section under spinal anesthesia. Ain-Shams J Anaesthesiol.

[19] Dehghanpisheh L, Azemati S, Hamedi M, Fattahisaravi Z (2021). The effect of 1-mg versus 3-mg granisetron on shivering and nausea in cesarean section:a randomized, controlled, triple-blind, clinical trial. Brazil J Anesthesiol. (English Edition).

[20] Sommariva S, Pongiglione B, Tarricone R (2016). Impact of chemotherapy-induced nausea and vomiting on health-related quality of life and resource utilization:A systematic review. Crit Rev Oncol/hematol.

